# CNN-HT: A Two-Stage Algorithm Selection Framework

**DOI:** 10.3390/e26030262

**Published:** 2024-03-14

**Authors:** Siyi Xu, Wenwen Liu, Chengpei Wu, Junli Li

**Affiliations:** 1 School of Computer Science, Sichuan Normal University, Chengdu 610068, China; xusiyi1999@163.com (S.X.); wenwenl18ctu@gmail.com (W.L.); chengpei.wu@hotmail.com (C.W.); 2 Visual Computing and Virtual Reality Key Laboratory of Sichuan, Sichuan Normal University, Chengdu 610068, China; 3 Key Laboratory of Land Resources Evaluation and Monitoring in Southwest (Sichuan Normal University), Ministry of Education, Chengdu 610068, China

**Keywords:** algorithm selection, convolutional neural network, exploratory landscape analysis, classification, feature selection, hypothesis testing

## Abstract

The No Free Lunch Theorem tells us that no algorithm can beat other algorithms on all types of problems. The algorithm selection structure is proposed to select the most suitable algorithm from a set of algorithms for an unknown optimization problem. This paper introduces an innovative algorithm selection approach called the CNN-HT, which is a two-stage algorithm selection framework. In the first stage, a Convolutional Neural Network (CNN) is employed to classify problems. In the second stage, the Hypothesis Testing (HT) technique is used to suggest the best-performing algorithm based on the statistical analysis of the performance metric of algorithms that address various problem categories. The two-stage approach can adapt to different algorithm combinations without the need to retrain the entire model, and modifications can be made in the second stage only, which is an improvement of one-stage approaches. To provide a more general structure for the classification model, we adopt Exploratory Landscape Analysis (ELA) features of the problem as input and utilize feature selection techniques to reduce the redundant ones. In problem classification, the average accuracy of classifying problems using CNN is 96%, which demonstrates the advantages of CNN compared to Random Forest and Support Vector Machines. After feature selection, the accuracy increases to 98.8%, further improving the classification performance while reducing the computational cost. This demonstrates the effectiveness of the first stage of the CNN-HT method, which provides a basis for algorithm selection. In the experiments, CNN-HT shows the advantages of the second stage algorithm as well as good performance with better average rankings in different algorithm combinations compared to the individual algorithms and another algorithm combination approach.

## 1. Introduction

Black-box optimization problems are a crucial part of the optimization research field [1], characterized by the absence of mathematical structures such as derivability, linearity, convexity, or even inaccessible objective functions. Many engineering problems can be viewed or represented as black-box optimization problems [2,3]. In recent years, several evolutionary algorithms have been developed to address these types of problems. These algorithms offer stochastic optimization techniques that do not rely on any knowledge about the mathematical nature of the problem [4,5,6,7,8,9]. Theoretical research, including the No Free Lunch (NFL) theorems, found that all algorithms, without revisits, have average performances in the case that the distribution of all the problems is uniform [10]. Additionally, empirical studies have consistently demonstrated that no single algorithm can consistently outperform others across various benchmark and real-world optimization problems. The No Free Lunch (NFL) theorem guides that the algorithm set should have diversity and the connection between problems and algorithms should be built. Consequently, different evolutionary algorithms should be employed to tackle different problem types. Therefore, it is crucial to select an appropriate combination of algorithms to solve a given black-box optimization problem.

Algorithm combination methods involve using a collection of multiple algorithms to solve a given black-box problem, which can be classified into two main categories: algorithm portfolio and algorithm selection. In the algorithm portfolio approach, each algorithm in the collection runs independently on the problem instance, and the best solution among all the algorithms is considered as the solution for the entire portfolio. An example of the algorithm portfolio approach is the population-based algorithm portfolios (PAPs) proposed in [11], where each algorithm is allocated a portion of the time budget and interactions between algorithm sets are facilitated through a migration scheme. On the other hand, the algorithm selection approach focuses on selecting the most suitable algorithm from a set of available algorithms to solve a given problem. For instance, Kerschke et al. [12] utilize various machine learning models, such as Support Vector Machines, to construct algorithm selection models. While algorithm selection has been extensively studied for discrete problems, it has only recently gained attention for black-box problems [13]. However, most existing research in this area has primarily relied on reliable machine learning models. In recent years, deep neural networks [14,15] have gained significant prominence and found applications in various fields, including natural language processing and speech recognition. Nonetheless, there is limited research on applying deep learning models to algorithm selection problems.

The algorithm selection problem framework, introduced in [16] by Rice, provides general guidance for designing algorithm selection systems and consists of four essential components. The first component is the problem space, in which which encompasses problems of various dimensions and categories, such as continuous and discrete. The second component, the characteristics space, is used to analyze the problem’s properties in terms of derivability, linearity, convexity, and other factors. The third component is the algorithm space, which goes beyond a simple collection of algorithms and emphasizes the need for complementary algorithms suitable for various problems. The fourth component is the performance space, which captures the interaction between problems and algorithms, ensuring that a suitable algorithm selection method produces better solutions with lower computational costs. Various algorithm selection methods have been proposed based on Rice’s framework. For instance, Bischl et al. [17] introduced a cost-sensitive one-sided regression algorithm selection model that leverages exploratory landscape analysis. However, existing algorithm selection methods can be considered as one-stage methods suitable for fixed problem spaces and algorithm spaces. When the problems or algorithms evolve and update, modifying the algorithm combinations requires retraining the entire algorithm selection model, resulting in significant time costs and limited scalability. In other domains, two-stage approaches [18] have been proposed to address the issue of non-updatable models. For example, in the field of big data analytics, Khan et al. [19] developed a two-stage framework based on Spark machine learning and long and short-term memory networks. This framework allows the updating of big data analytics models by modifying specific components, enabling adaptation to the continuous growth and rapid advancement of big data. Liu et al. [20] and Zhang et al. [21] both utilized a two-stage method in addressing the issues of fresh product supplier selection and lane detection, respectively, showcasing their adaptable nature. However, in the field of algorithm selection, there is currently no such two-stage approach to address the scalability problem of models.

Following Rice’s algorithm selection structure of [16] to include the components of problem space and characteristic space, in order to identify the appropriate algorithms for a given problem, it is essential to deconstruct the problem space and analyze its characteristics. One of the techniques to find the characteristics of the problem landscape is Exploratory Landscape Analysis (ELA) [22,23], which utilizes numerical values to quantify and indicate the features of the problem landscape. Many ELA features have been proposed in recent years. Mersmann et al. [24] discuss eight attributes that characterize the complexity of an optimization problem, such as the global structure of the problem and multimodality. Many ELA features have been applied to algorithm selection. Bischl et al. [17] propose a cost-sensitive one-sided regression algorithm selection model based on exploratory landscape analysis. The paper focuses on machine learning models and for the feature space just uses multiple low-level features without focusing on whether different ELA features can accurately identify black-box problems. The issue of selecting the most appropriate and concise feature application in algorithm selection from a large number of ELA features is an important one.

The relationship between the algorithm space and the performance space is contingent on the objective of algorithm selection. Depending on the objective, researchers employ various methods for algorithm performance evaluation. Some algorithm selection studies aim to select the algorithm that solves the given problem more efficiently, utilizing a process-oriented evaluation method. Kerschke et al. [25] propose a structured approach for the variation of solver time across runs. They use the speed at which an algorithm solves a given problem as a criterion for evaluating how well an algorithm performs. The goal of some algorithm selection studies is to select the algorithm that finds the optimal value for a given problem, which is a result-oriented evaluation method. Tian et al. [26] use the ranking of the number of times an algorithm obtains the optimal solution to a problem as the performance of the algorithm. They use the numerical value size of the optimal value obtained by the algorithm to solve the given problem as a criterion for evaluating the performance of the algorithm.

In this paper, we focus on recommending the suitable algorithm for a given continuous black-box optimization problem, where a two-stage framework is applied. In the first stage of identifying the problem, we implement problem classification by combining Convolutional Neural Networks (CNNs) with ELA features to identify the unknown black-box optimization problem as the pre-defined problem class. In order to reduce the input of CNN and provide more effective information, we apply the feature selection techniques to simplify redundant features. In the second stage of selecting the algorithm, we propose an algorithm selection strategy to select the appropriate algorithm for the known problem type. The algorithm selection strategy uses statistical hypothesis testing results to recommend the algorithm. Applying hypothesis testing makes the difference in performance between algorithms significant, thus ensuring that the results are statistically reproducible and stable. Therefore, the four main contributions of this paper are summarized as follows:

- This paper presents a novel algorithm selection framework named CNN-HT, which follows a two-stage approach. In contrast, existing algorithm selection methods, based on the structure presented in [16], typically employ a one-stage approach involving a regression or classification model to establish the relationship between problems and algorithms. However, one-stage approaches incur high computational costs when adapting to changing problems and algorithms, as the classification models need to be retrained in such instances. So the algorithm has the advantage of being able to adapt to different combinations of algorithms without the need to re-train the whole model, simply by making modifications in the second stage. Experiment 4.4 demonstrates CNN-HT’s adaptability to algorithm sets of different sizes.
- The paper demonstrates that deep learning CNN is the most suitable classification model for identifying problem classes compared to other classification models. In Experiments 4.2 and 4.3, CNN, Random Forest, and Support Vector Machine (SVM) are used as classification models, with CNN exhibiting the highest accuracy.
- Feature selection techniques are applied as a preprocessing step in problem classification, reducing redundant features and saving computational costs for training the classification model, as the model has fewer parameters to train with reduced input. Experiment 4.3 indicates that the selected features as input achieved higher accuracy compared to the initial 169 features and randomly selected 19 features.
- The CNN-HT method outperforms individual algorithms within the algorithm set and another algorithm combination, PAP, which is supported by Experiment 4.4.

The rest of the paper is organized as follows: The related work in Section 2 is reviewed and summarized. Section 3 presents the framework and details of the new algorithm selection. In Section 4, three experiments are conducted to investigate the performance of our approach. Finally, the paper is summarized and future work is provided.

## 2. Related Works

### 2.1. Exploratory Landscape Analysis

Selecting an algorithm for a continuous optimization problem is challenging due to its complexity and limited information on the problem. The characteristics space, the second component of the algorithm selection structure, is defined by a set of metrics that offer insights into the problem’s complexity. For single-objective continuous optimization problems, the feature generation process is known as exploratory landscape analysis. This process, as described in [22,23], aims to characterize the landscape using a set of numerical features.

Advanced feature characterization problems, as introduced in [24], are derived from expert studies and encompass metrics such as the level of modality and separability. In [27], the features used to describe the problem landscape are categorized into five groups, including metamodel features and convexity features. Additionally, new landscape features have been introduced for algorithm selection, such as those described in [28] for constrained optimization problems and by Shirakawa and Nagao in [29] for local landscape features. These features can be generated for black-box optimization problems by sampling the problem and then computing them.

In this paper, the Flacco platform [30] is utilized to compute features for quantified problem landscapes. Flacco is a platform-independent web application that employs a graphical user interface to calculate over 300 exploratory landscape analysis features in a uniform manner.

### 2.2. Feature Selection

Feature selection is a common data preprocessing technique in pattern recognition, machine learning, and data mining [31,32,33,34,35,36]. It involves choosing a subset of features from the full set to enhance the model’s accuracy and stability. In practical machine learning applications, a higher number of features increases the time required for analysis and model training [37,38]. Additionally, a larger number of features can lead to a “dimensional catastrophe”, resulting in a more complex model with reduced generalization ability. Feature selection addresses these issues by eliminating irrelevant or redundant features, ultimately improving accuracy, stability, and reducing runtime [39].

In our work, we employ the naive forward attribute reduction based on the neighborhood rough set (NFARNRS) feature selection algorithm [40], comprising four steps. The first step involves the generation process, which searches for a subset of features for the evaluation function. The second step is the evaluation function, which assesses the quality of the feature subset. The third step is the stopping criterion, which determines when to halt the search based on the evaluation function. The final step is the result validation, which verifies the efficacy of the selected feature subset on the validation dataset. Conceptually, for a problem with multiple classes, the algorithm seeks a feature subspace with minimal overlap between classes.

By employing a feature selection strategy, we reduce the feature dimensionality and improve the classification performance. Eliminating abundant features simplifies the model input, thus reducing the computational cost.

### 2.3. Convolutional Neural Network

A Convolutional Neural Network [14,15,41,42,43] is a very important neural network structure in deep learning. It shows powerful capabilities in image and picture processing, video processing, audio processing, and natural language processing [44,45,46]. However, there are few studies that apply deep learning to algorithm selection. In [47], the authors of this paper use deep neural networks to construct algorithm selection models for continuous optimization problems. The number of input parameters in their network is small and the amount of their training data is also small, so the method does not show the capability of deep neural networks. In [48], this paper also uses Convolutional Neural Networks to construct algorithm selection models for Boolean satisfiability (SAT) problems, and the results in the paper show that the algorithm selection models perform well. However, the input to the Convolutional Neural Network in the paper is a SAT problem that can be described in a text file. For SAT problems that cannot be described by text files, the algorithm selection model is not solvable. In [49], the authors construct an algorithm selection model using a Convolutional Neural Network. In this paper, we extract the landscape information from the optimization problem and save the information as a two-dimensional image as the input to the convolutional neural network.

This paper introduces the utilization of a Convolutional Neural Network (CNN) for accurate classification and prediction of black-box optimization problems. The CNN classification model demonstrates its effectiveness in algorithm selection, as it not only achieves proficient problem classification but also enhances the performance of the algorithm selection model we developed.

### 2.4. Hypothesis Testing

Hypothesis testing [50] is a statistical inference method. It is used to identify whether the sample-to-sample differences are caused by sampling error or by essential differences. Depending on the number of samples, the test is different. The Mann–Whitney U test [51] is a nonparametric statistical test that is often used to test for significant differences between the results of two samples. Significance level α is a predefined parameter. Given a *p*-value of ρ, the output of the u-test, if ρ≤α, it proves that the two samples are significantly different; otherwise (ρ>α), it proves that there is no significant difference between the two samples. The Kruskal–Wallis test [52] is often used to test whether there is a significant difference between three or more sample results. Hypothesis testing is widely used in evolutionary algorithms. In [8,53,54], the method shows a UE/UD problem for a target algorithm, using statistical tests to determine a significant difference between the target algorithm and other algorithms.

## 3. CNN-HT

**Figure 1:**
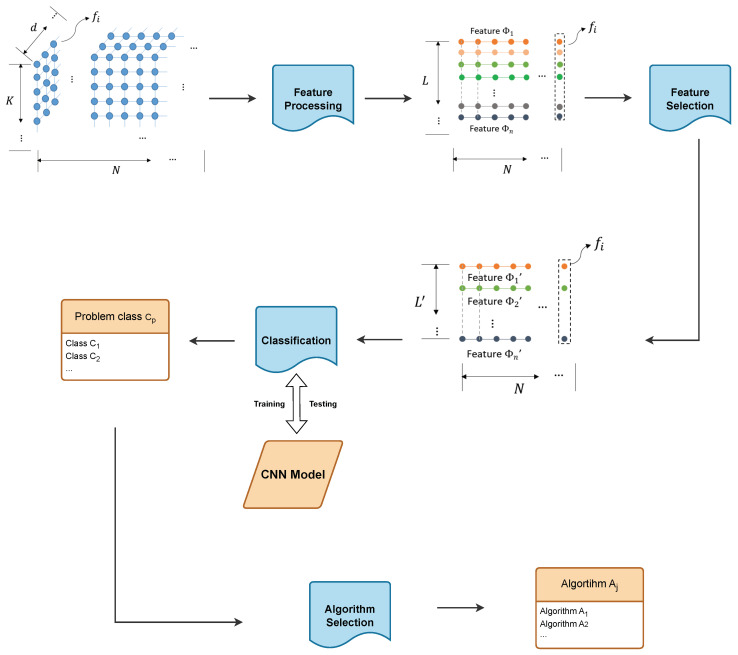
Flowchart of CNN-HT. It starts with sampling the points of the problem, performing feature processing to obtain the features of the problem (ELA features), then performing feature selection to obtain the simplified features, passing the simplified features through the classification model to obtain the problem label, and obtaining the recommended algorithm for a given problem according to the algorithm selection strategy.

We consider a black-box optimization problem class with N problem instances. $P≜\{f_{1},f_{2},\ldots ,f_{N}\}$ is given; for each problem, $f_{i}$ for i=1,2,…,N, $f_{i}:R^{d}\to R$ with input $x\in R^{d}$. The objective is to select a suitable algorithm for a randomly selected problem $f_{i}$ when i∈{1,2,…,N} to find the optimum value $f_{i}^{*}\left(x\right)$. We assume that there is an algorithm set $A≜\{a_{1},a_{2},\ldots ,a_{M}\}$ to solve P. In our proposed algorithm selection framework, we intend to offer a method mapping from problem $f_{i}\in P,i\in \left\{1,2,\ldots ,N\right\}$ to algorithm $a_{j}\in A,j\in \left\{1,2,\ldots ,M\right\}$. The structure of the algorithm framework is shown in Figure 1.

The first step is to estimate the sampling space for randomly selected *N* problem instances from problem class P. We use the Latin Square sampling approach [55] to sample an input variable matrix with a size of *K* ∗ *d*, where *K* is the number of samples. We assume the input variable matrix $X_{i}=\left\{x_{1},x_{2},\ldots ,x_{K}\right\}$ and the corresponding fitness values are $F_{i}=\left\{f_{i}\left(x_{1}\right),\ldots ,f_{i}\left(x_{K}\right)\right\}$ for each problem instance $f_{i}$ for i∈I with I={1,2,…,N} as the selected indexes from {1,2,…,N}. Thus, the sampling space for *N* problem instances can be represented as $S=\{\left(X_{i},F_{i}\right):i=1,2,\ldots ,N\}$, where the different indexes of *X* indicate random sampling points for each problem instance $f_{i}$.

In the second step, raw searching space *S* is transferred to feature space $S_{\phi }$ to extract important information about the landscape. We assume ϕ is a function to calculate the numerical value of one ELA feature with the input $\left(X_{i},F_{i}\right)$. We take ϕ(·) to represent it. In the ELA feature study, common features include convexity, distance, etc. [13]. We assume that there is a set of feature function $G=\{\phi_{1}\left(\cdot \right),\phi_{2}\left(\cdot \right)\ldots ,\phi_{L}\left(\cdot \right)\}$ to evaluate the landscape of a randomly problem instance. We assume *L* feature functions are applied to extract the feature. For any $f_{i}$ and i∈I, the information of raw sampling data $\left(X_{i},F_{i}\right)$ can be transferred to feature vector $\Phi_{i}=\left\{\phi_{j}\left(X_{i},F_{i}\right):j=1,2,\ldots ,L\right\}$. Thus, the feature space for the *N* problem can be represented as $S_{\phi }=\left\{\Phi_{1},\ldots ,\Phi_{N}\right\}$. Note that $\Phi_{i}$ is a *L* ∗ 1 vector, and *L* is the feature size. With larger *L*, more features are utilized to describe the problem instance according to the sampling points.

The following step of feature selection is to purge the redundant features and retain the most important ones. In terms of analyzing the feature data, some of the features are correlated. In the next step, the features are applied to classify the problem, and the correlated feature as the input may lead to bias in the classification model. To address this problem, we utilize the forward algorithm [40] to clean the features and reduce correlation among them. The step removes the most correlated feature before the performance degenerates. This step reduces the number of features from *L* to $L^{\prime }$. Correspondingly, the feature space is also reduced from $S_{\phi }$ to $S_{\phi }^{\prime }$.

The third step is to apply a classification model to identify the group label of the given problem. We assume that *N* problem instances can be divided into *Q* categories according to the different properties of problems. The categories are defined as $C≜\{c_{1},c_{2},\ldots ,c_{Q}\}$. There is a mapping relationship P→C to indicate the category label of each problem instance. In our paper, we utilize CNN as the classification model, with input space $S_{\phi }$ or $S_{\phi }^{\prime }$. And the output, which is the label of categories, is defined as the class number of BBOB. During the training process, the CNN model continuously adjusts the weights and biases to minimize the loss function through the backpropagation algorithm. In the training phase of the CNN, the objective is to minimize the cross-entropy loss function between predicted label $y_{i}$ of the problem and real label $\hat{y_{i}}$. The equation of the loss function is $Loss=-\sum_{n}^{i=1}y_{i}\log\hat{y_{i}}$

**Figure 2:**
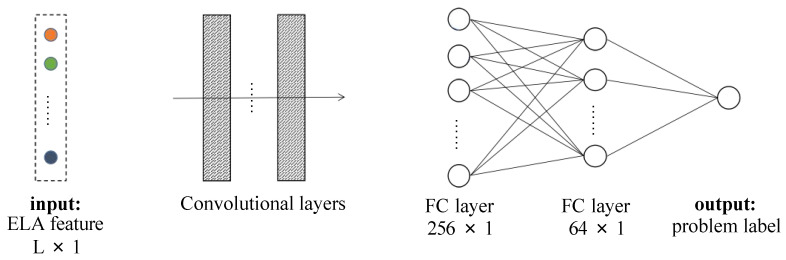
Structure of CNN. The input to the CNN is the ELA features of the given problem instance; initially—L = 169 and after feature selection—L = 19. The input to the CNN is the ELA features of the given problem instance; initially—L = 169 and after feature selection—L = 19. The output is the class label of the problem. For the BBOB problem, the output is one of the labels classified into 24 classes.

Therefore, in the testing phase, when an unknown problem occurs as the input, the model can accurately classify the class of the problem. It should be noted that the classification model can be replaced in the general framework for more purposes. We adopt the CNN model as an efficient classification model for unknown problems. The structure of the CNN model is shown in Figure 2.

The last step is to estimate the mapping relationship: C→A. Assuming that there are *N* problems that can be divided into *Q* categories, we let *M* algorithms independently solve the *N* problem instances for *W* times. If it is assumed that algorithm $a_{j}$ outperforms any other algorithms from A on problem class $C_{p}$, $a_{j}$ is the most suitable to solve $C_{p}$. Also, the performance can be estimated statistically by the Mann–Whitney U-test. A resulting matrix of M∗N∗W is recorded after all *M* algorithms solve *N* problems. The statistical significance of each pair of the algorithm performance is calculated, and the superior algorithm is marked. Following the results of the Mann–Whitney U-test, the corresponding relationship between algorithms and problem classes is estimated.

In practical use, when random problem instance $f_{t}$ is selected from P, where t∈{1,2,…,N}∖I, meaning that $f_{t}$ is not involved in establishing the mapping relationship between P→C and C→A, our algorithm selection framework is capable of providing recommendations for the most suitable algorithms to solve $f_{t}$ based on the constructed framework.

Figure 1 demonstrates the framework of the methodology. First, we conduct Latin hypercube sampling on the problem to obtain a set of sample points and calculate the corresponding fitness values for each sample point. Next, the data are processed through the flacco platform for feature extraction, resulting in ELA features for the problem. Subsequently, the ELA features are processed using a feature selection algorithm to obtain a reduced feature set. These features are then input into a classifier (Convolutional Neural Networks) for problem classification, resulting in problem labels. Finally, based on our algorithm selection strategy using hypothesis testing, an appropriate algorithm is chosen for the given labels. The structure of the CNN model is shown in Figure 2.

## 4. Experimental Results

### 4.1. Data Description

In the next experiments, we evaluate the performance of our method using the BBOB problems. Black-Box Optimization Benchmarking (BBOB) is a public benchmark set for black-box optimization problems. It encompasses a representative variety of simple and complex problems, so BBOB is chosen as the experimental problem set. BBOB problems can be divided into 24 problem classes. For each class, different problem instances can be generated by random transformations and rotations. The search space of the instances is ${\left[-5,5\right]}^{D}$, where D is the problem dimension. We investigate examples of BBOB problems on D = 2, 5, 10 and 20. In our work, we use the Python language to complete the experiments. Our work is implemented on an Intel(R) HD Graphics 520 graphics card.

In our work, we use a 1D CNN to study the classification ability of ELA features for the BBOB problem. The 1D CNN architecture is implemented by the Python language on the keras framework. The detailed setup of the 1D CNN architecture is shown in Table 1. The 1D CNN architecture consists of three sets of convolutional layers, a fully connected layer and a soft-max layer. We set the number of training epochs to 40, where one epoch indicates that the network is trained once. We set the batch size to 1. In the CNN, we first train the network and use test instances to evaluate the performance of the network after completing the training.

### 4.2. Results of Problem Classification

In the first experiment, we investigate the classification ability of our method on instances of BBOB functions with D = 2, 5, 10 and 20. In this section, the experimental Convolutional Neural Networks are compared with two machine learning methods, Support Vector Machines and Random Forests, to show the advantages of Convolutional Neural Networks as classifiers. The total evaluation budget for the experiments is 10,000 D.

The first phase of the experiment involves obtaining Exploratory Landscape Analysis (ELA) features for BBOB problems through computation on the flacco platform. Flacco serves as a comprehensive platform, consolidating various ELA features proposed by researchers, enabling the computation of over 300 ELA features for a single optimization problem. ELA features are numerical features used to describe the problem landscape. Each ELA feature can describe some properties of the problem. Given that BBOB problems are black-box in nature, we employ Latin Hypercube sampling to obtain ELA features by sampling the problem instances. Each BBOB function instance is sampled at 1% of the total evaluation budget. For example, with D = 20, the number of BBOB function instances sampled is 2000. Fitness values are determined based on the sampling points, and ELA features are computed from these fitness values to create the feature dataset for the BBOB problem. To mitigate the impact of redundant and excessively large ELA feature values on classification, we perform rough feature selection, discarding features with identical values across all functions or with excessive data. This processing results in 169 Exploratory Landscape Analysis features per instance, representing each problem instance with 169 values. Table 2 shows some of the ELA feature values in the selected BBOB problem dataset.

The second phase of the experiment involves utilizing these ELA features as inputs for the classification and prediction of BBOB problems using a 1D CNN. The 1D CNN is trained to recognize unknown black-box problems and classify them into 24 known classes. Concurrently, a comparative experiment is conducted between 1D CNN, Random Forest (RF) [56], and Support Vector Machine (SVM) [57] for BBOB problem classification. Support Vector Machine classification is based on solving a separating hyperplane that correctly divides the training dataset and maximizes the geometric interval. The experimental parameters of the Support Vector Machine are set to a regularization factor of 2, the kernel function uses the radial basis function (RBF), the multi-categorization strategy parameter is set to ”ovo“, which means dividing the categories between two by two, and all other parameters are used as default settings. Random Forest experimental parameters are set to select 100 trees to construct a Random Forest, information entropy is chosen to find nodes and branches, and default settings are used for all other parameters.

In all three classifiers, the inputs comprise the 169 exploratory landscape analysis features from the flacco platform, and the outputs are the category labels of the BBOB problem, categorized into 24 classes, each generating 100 instances. Thus, there are 100 × 24 = 2400 instances in total. We use 70% of the instances for training and 30% for testing. Given an unknown instance in the test set, the trained model is used to predict its problem class. In total, there are 24 predictions. To avoid randomness, for each classifier, we repeat training and testing five times to obtain five well-trained models. We choose the test results obtained by the model at the median as the reliable results.

Table 3 shows the classification results for instances using the three classifiers with D = 2, 5, 10 and 20. For the cases of D = 2, 5, 10 and 20, the average classification accuracies of using a 1D CNN combined with Exploratory Landscape Analysis features to classify the BBOB problem are 99.97%, 96.78%, 95.71% and 93.79%; the average classification accuracies using Random Forest as a classifier are 94.3%, 94.12%, 93.3%, 91.22%; the average classification accuracies using Support Vector Machine as a classifier are 96.25%, 93.1%, 93.54%, 91.56%. All classifiers achieve accuracies above 90%, indicating their ability to correctly classify most problems. Notably, 1D CNNs consistently outperform Random Forests and Support Vector Machines, suggesting that using 1D CNNs as classifiers results in more accurate BBOB problem classification.

### 4.3. Results of Feature Selection

In the second experiment, we focus on performing feature selection for exploratory landscape analysis (ELA) features. In this section, some comparative experiments are used to demonstrate the advantages of feature selection in not only improving classification performance but also reducing feature dimensionality. Although the initial experiments successfully classify the BBOB problem with 169 features, there are redundant and invalid features in this set. These redundancies and invalidities not only impact classifier accuracy but also contribute to increased computation time and complexity. Consequently, our aim is to identify effective and fewer ELA features without degrading the accuracy of the categorized BBOB problem.

In the first step of the experiment, we perform ELA feature selection for each dimension separately. Utilizing the NFARNRS algorithm, we derive four sets of optimized ELA features corresponding to dimensions D = 2, 5, 10, and 20. The input to the feature selection experiment is 169 exploratory landscape analysis features obtained by sampling the BBOB problem in the first experiment. The NFARNRS algorithm processes this feature set to yield a subset with the highest dependency, possessing the same discriminative power as the original 169 ELA features without any redundancy. In dimensions D = 2, 5, 10, and 20, the NFARNRS algorithm selects 14, 22, 13, and 21 features, respectively. The subsets of features we obtain are all features that maximally distinguish the problem. These features are features with relevance, based on which high-level features can be obtained that have a strong impact on the problem and thus successfully characterize the problem. For example, in D = 2, we choose ELA convexity features, which describe the convexity of the function. We choose the ELA y-distribution feature, which describes the skewness and kurtosis of the objective function values. The convexity of the function and the kurtosis of the distribution are estimated, and these two features are used as indicators of multimodality. The f1 Sphere function in the BBOB function does not have a multimodal form, the f3 Rastrigin separable function is a high multimodal function form, and the f9 Rosenbrock rotated function is a low multimodal function form. These three functions can be clearly distinguished by these two features. So we choose such features that can more clearly distinguish the BBOB problem as a smaller ensemble of features to characterize the problem. By classifying the results of the BBOB problem with a subset of features after feature selection, we investigate whether the features are extracted effectively.

The second step of the experiment involves integrating the previously acquired feature sets from different dimensions into a unified feature set. While these feature sets are dimensionally specific and represent the most relevant features for each dimension, they lack universality. To address this, we integrate the four dimensional feature sets by selecting their intersection, resulting in a new feature set applicable to D = 2, 5, 10, and 20. This consolidated feature set comprises 19 ELA features, offering a representative and general solution. Table 4 provides detailed descriptions of these 19 features, including ELA convexity features, linear model features, and others. These features are also highly relevant and based on key features of the problem landscape, such as the degree of global structure or the number of local optimizations, both of which have been shown to have a large impact on differentiating problems. Consequently, this new feature set allows for a more effective and concise description of the BBOB problem. We examine the effectiveness of feature selection by inputting a new set of features into the classifier to classify the problem.

As shown in Table 5, we investigate the classification ability of feature sets with different dimensions for the BBOB problem. Initially, we assess the classification performance of the feature set after feature selection. As shown in the first row of Table 5, the classifier achieves classification accuracies above 98% for D = 2, 5, 10, and 20. This outcome substantiates the effectiveness of our feature selection in identifying representative features from a myriad of ELA features, thereby enhancing the accuracy of problem classification.

Three comparison experiments are set up in this experiment. Comparison Experiment 1 aims to verify the correctness of feature selection. In this experiment, 1D CNN, Random Forest, and Support Vector Machine classify the BBOB problem using the dataset post-feature selection. As depicted in Comparison Experiment 1 in Table 5, the results indicate high classification accuracy for all three classifiers, affirming that the new feature set (comprising 19 features) accurately classifies the problem. Moreover, the superiority of 1D CNN over Random Forest and Support Vector Machine underscores the rationale for choosing 1D CNN as the problem classifier, as it exhibits higher accuracy in BBOB problem classification.

Comparison Experiment 2 focuses on the necessity and effectiveness of feature selection. Comparison Experiment 2 is the experiments of classifying the BBOB problem using 19 randomly selected features and the initial 169 features. As shown in Table 5, the first row of Contrast Experiment 2 is the accuracy result of classifying the BBOB problem for the randomly selected set of 19 features. The feature set of 19 randomly selected features is the feature set obtained by selecting multiple features from 169 features in a completely random manner. The second row of Comparison Experiment 2 shows the classifier’s accuracy results for the BBOB problem of classifying an initial feature set containing 169 features. The results show that the classification accuracy of the feature selection experiment is higher than the classification accuracy of Comparison Experiment 2. This demonstrates the strong representativeness and interpretability of the feature set obtained from feature selection. With random selection as the benchmark, feature selection not only ensures or improves classification accuracy but also reduces the number and dimension of features, simplifying the dataset.

Comparison Experiment 3 delves into the generality and universality of feature selection by comparing the classification abilities of feature sets obtained by selecting features of different dimensions and those integrated after selection for the BBOB problems. As shown in Comparison Experiment 3 in Table 5, the first, second, third, and fourth rows present classification results using the feature set after feature selection for D = 2, 5, 10, and 20, respectively. Comparison results show a decrease in classification, which still results in a 98% rate. This indicates the meaningfulness of our integration process, which amalgamates dimension-specific feature sets into a universal feature set, demonstrating that the new feature set is indeed universal.

### 4.4. Performance Analysis of Algorithm Selection

The third experiment focuses on evaluating the performance of the CNN-HT algorithm selection method. The previous experiments successfully showcased the accurate problem classification achieved through ELA features and 1D CNN. In the following analysis, we compare the performance and scalability of the CNN-HT method with that of a single optimization algorithm and the PAP algorithm combination method. Additionally, as a two-stage approach, we apply CNN-HT with two different algorithm sets while utilizing the same configuration of the trained CNN classification model discussed in Section 4.2 to demonstrate the adaptability of our method to the different algorithm set settings.

We commence by conducting experiments on algorithm selection strategies, focusing on identifying the optimal combination of algorithms. The algorithm portfolio necessitates the selection of complementary algorithms aiming to solve a broader spectrum of problems with a reduced set of algorithms. Adhering to this principle, we choose five well-established algorithms with diverse evolutionary principles: Composite Differential Evolution (CoDE) [58], Evolution Strategy with Covariance Matrix Adaptation (CMA-ES) [59], the Squirrel search algorithm (SSA) [60], the adaptive mechanism in Success-History based Adaptive Differential Evolution (L-SHADE) [61], and the Zebra Optimization Algorithm (ZOA) [62]. CMA-ES is a very classical algorithm for solving the black box problem, and ZOA and SSA are both very new algorithms; these algorithms show good complementarity in solving the BBOB problem. Therefore, the algorithm pool is set to A = {CoDE, CMA-ES, SSA, L-SHADE, ZOA}. We use the default parameters introduced in these evolutionary algorithm references (i.e., [58,59,60,61,62]). The algorithm selection strategy is proposed based on hypothesis testing. Our experimental setup is the 24 BBOB function problem with D = 10 and a total evaluation budget of 10,000 D. For each of the 24 BBOB functions, we select 5 instances for a total of 120 instances. Because the algorithm search is randomized, the four algorithms in the algorithm portfolio are run 10 times on each instance. The algorithm with the lowest fitness result obtained from the four algorithm runs is taken as the optimal algorithm to obtain the performance of the four algorithms on each problem. The algorithms are then ranked based on their performance for each of the 24 BBOB functions. Initially, we select the top-ranked algorithm and perform a Mann–Whitney U test on the top- and bottom-ranked algorithms to identify significant differences. If no significant difference is found between the top-ranked and second-ranked algorithms, we add the second-ranked algorithm to the selection set. Subsequent hypothesis testing is conducted iteratively until the last algorithm participates. This statistical testing-based algorithm selection strategy, employing the Mann–Whitney U test, significantly diminishes the randomness of algorithm performance, ensuring the accuracy of our recommended algorithms.

As shown in Table 6, we obtain the set of recommended algorithms for each BBOB problem. In instances where more than one algorithm is selected for a problem, we adhere to the random principle and randomly choose one selected algorithm as the recommended algorithm. For example, in the case of the f7 function, where CoDE, SSA, and L-SHADE are selected, we randomly choose one algorithm from the selection as the recommended algorithm. This algorithm selection strategy demonstrates that for each of the 24 BBOB problems, every algorithm has the opportunity to be the optimal one. Different algorithms exhibit strengths in solving distinct classes of problems, indicating that the set of algorithms we employ is both small and complementary.

Moving on, we scrutinize the performance of the CNN-HT algorithm selection method by comparing it with other algorithms. The comparison algorithms include five single optimization algorithms (CoDE, CMA-ES, SSA, L-SHADE, ZOA) and the population-based algorithm portfolio (PAP) [11]. The PAP method also uses multiple algorithms as a combination to solve the problem, so we choose PAP as the comparison algorithm. To maintain consistency, the algorithm portfolios for the PAP approach in these experiments also consist of CoDE, CMA-ES, SSA, L-SHADE and ZOA. For the CNN-HT algorithm selection method, the experiments are set up with 24 BBOB functions, D = 10, and a total evaluation budget of 9900 D. We use 1% of the evaluation budget for classification and 99% of the evaluation budget for search. For the 24 BBOB functions, we select 5 test instances, and there are 120 instances in total. Using a trained network, one optimization algorithm is recommended for each test instance from the algorithm set, and the CNN-HT algorithm selection method recommended algorithm is run 10 times on each instance separately. For the experimental setup of the comparison algorithms, the total evaluation budget is 10,000 D and the other experimental setups are the same as above. In the experiments comparing the performance ranking of the CNN-HT method with the comparison algorithms, we test for significance using the Kruskal–Wallis test and the multiple comparison test with *p*-value = 0.05.

Table 7 shows the performance ranking of the CNN-HT algorithm selection method and the comparison algorithms. Table 7 also shows the number of CNN-HT algorithm selection methods that significantly improve the comparison algorithms by hypothesis testing on the corresponding problems. The results in Table 7 reveal that the average ranking of the CNN-HT algorithm selection method is 1.291, making it the top-ranking method compared to the comparison algorithms. The CNN-HT algorithm selection method either outperforms or matches the performance of the five single optimization algorithms across most problems, underscoring its effectiveness. Moreover, the CNN-HT algorithm selection method surpasses the PAP algorithm portfolio, suggesting not only its efficacy but also an enhancement in performance over other algorithm combination methods. For the majority of problems, our method excels at recommending the most suitable optimization algorithm for a given problem instance.

We extend the application of the algorithm recommendation strategies derived for D = 10 to D = 2, 5, and 20, respectively. In these dimensions, we compare the CNN-HT algorithm selection method with five single optimization algorithms (CoDE, CMA-ES, SSA, L-SHADE and ZOA) and the population-based algorithm portfolio (PAP). The experimental setup remains consistent with that of D = 10. As shown in Table 8, Table 9 and Table 10, the rankings for D = 2, 5, and 20 are 1.416, 1.33, and 1.416, respectively. These results consistently demonstrate that the CNN-HT algorithm selection method maintains a high ranking, outperforming or matching the single optimization algorithms and the PAP algorithm portfolio across most problems. This underscores the effectiveness and scalability of our algorithm selection model.

We further apply the CNN-HT algorithm selection method to different combinations of algorithms. We only need to change the second part of the method to change the correspondence between the problem and the algorithm. We use four well-established algorithms with different evolutionary principles, namely Artificial Bee Colony (ABC) [63], Self-adaptive Differential Evolution (SaDE) [64], Evolution Strategy with Covariance Matrix Adaptation (CMA-ES) [59], and the Global-Local algorithm (GL-25) [65]. Therefore, the algorithm pool is set to A = {ABC, SaDE, CMA-ES, GL-25}. For different combinations of algorithms, we conduct experiments on the BBOB function with D = 10. The experimental setup is the same as above. Table 11 shows the performance rankings of the CNN-HT algorithm selection method, the four single optimization algorithms, and the PAP method under a different algorithm portfolio, as well as the number of CNN-HT algorithm selection methods that perform significantly better than the comparison algorithms using hypothesis testing on the corresponding problems. The experiments demonstrate that the CNN-HT algorithm selection method maintains a high ranking, with the recommended algorithms consistently outperforming or equaling the comparison algorithms across most problems. This highlights the fact that our approach is not only not limited to algorithm combinations, but also allows for easier updating of algorithms.

## 5. Conclusions

Our paper introduces a novel algorithm selection model called CNN-HT. This model operates in two stages, with the first stage utilizing a CNN as the classification model to classify the problem. Feature selection techniques are employed to select the most appropriate features for enhancing the accuracy of the classification. In the second stage, an algorithm selection strategy based on hypothesis testing is employed to recommend the optimal algorithm based on the classification of the problem. With this approach, our CNN-HT model can effectively select algorithms for solving intricate black-box optimization problems.

Our research encompasses a series of experiments aimed at assessing the efficacy of our approach. The initial experiments focus on evaluating the ability of the first phase of CNN-HT to categorize BBOB problems. We compare the CNN model used in this work with other machine learning models as classifiers. The results show that CNN demonstrates an advantage in classifying BBOB problems.

In the second experiment, we investigate the necessity and effectiveness of feature selection in the CNN-HT algorithm selection framework. With different numbers of ELA feature input classifiers, we compare a CNN with other machine learning methods as classifiers, both of which demonstrate the advantages of CNNs used for problem classification. Comparing the classification of the initial ELA features (169) with that of the features after feature selection (19), the results show that feature selection not only improves the classification performance, but also reduces the feature dimensionality, the time to compute the features and the computational cost of training the model.

Lastly, the third experiment involves a comparison between the performance of CNN-HT, single optimization algorithms, and the PAP algorithm selection method. The results reveal that our algorithm selection model surpasses both the single optimization algorithms and the PAP algorithm selection method in identifying the most suitable algorithm for solving the BBOB problem. Furthermore, our method exhibits adaptability to different algorithm set configurations.

We intend to expand our research in future work. While our experiments are limited to the BBOB problem in dimensions 2, 5, 10, and 20, we believe the CNN-HT algorithm selection model can be applied to higher dimensions of the BBOB problem. Additionally, while we only test CNN-HT on the well-known and widely accepted BBOB problem, we believe that our algorithm selection model has broad applicability to other problems. Therefore, in future work, we plan to focus on applying CNN-HT to a wider range of practical problems beyond the BBOB problem. Our goal is to generalize CNN-HT as an algorithm selection model for comprehensive benchmark problems and practical applications.

## Figures and Tables

**Table 1:** Parameter settings of 1D CNN architectures.

Group	Layers	Kernel Size	Stride	Output Channel
Group 1	Conv3-16	3	1	16
	Conv3-16	3	1	16
	Max3	3	1	16
Group 2	Conv3-64	3	1	64
	Conv3-64	3	1	64
	Max3	3	1	64
Group 3	Conv3-64	3	1	64
	Conv3-64	3	1	64
	Max3	3	1	64
	FC-64	1	1	64
	Softmax	1	1	24

**Table 2:** The partial dataset of ELA features for the BBOB problem with D = 2.

Feature Name	Feature Name in Flacco	F1	F2	F3
cell mapping features	cm_angle.dist_ctr2best.mean	2.77	2.63	1.94
gradient homogeneity features	cm_grad.mean	0.59	0.61	0.36
ELA curvature features	ela_curv.grad_norm.max	14.9	8,909,220.50	639.14
ELA y-distribution features	ela_distr.number_of_peaks	1.00	4.00	5.00

The table shows the three ELA feature values computed on one instance of the BBOB problem for F1, F2, and F3. The table is a partial sample from the dataset. The actual one instance has 169 feature values.

**Table 3:** The accuracy of classifying the BBOB instances with D = 2, D = 5, D = 10 and D = 20 into 24 problem classes using 1D CNN, Random Forest and Support Vector Machine.

	D = 2	D = 5	D = 10	D = 20
1D CNN	99.97%	96.78%	95.71%	93.79%
SVM	96.25%	93.1%	93.54%	91.56%
RF	94.3%	94.12%	93.3%	91.22%

**Table 4:** Summary of features obtained by feature selection, describing the meaning of sub-features of feature classes for classification and prediction of BBOB functions.

Feature Name	Feature Name In Flacco	Description
ELA convexity features	ela_conv.conv_prob	percentage of convexity
ELA convexity features	ela_conv.lin_dev.orig	average (original) deviation between the linear combination of the objectives and the objective of the linear combination of the observations
ELA curvature features	ela_curv.grad_scale.lq	aggregations (lower quartile) of the ratios between biggest and smallest (absolute) gradient directions
ELA curvature features	ela_curv.grad_scale.sd	aggregations (standard deviation) of the ratios between biggest and smallest (absolute) gradient directions
ELA y-distribution features	ela_distr.costs_runtime	number of features and runtime (in seconds) which were needed for the computation of these features
ELA local search features	e1a_local.best2mean_contr.orig	each cluster is represented by its center; this feature is the ratio of the objective values of the best and average cluster
ELA local search features	ela_local.best2mean_contr.ratio	each cluster is represented by its center; this feature is the ratio of the differences in the objective values of average to best and worst to best cluster
ELA local search features	ela_local.fun_evals.lq	aggregations (lower quartile) of the performed local searches
ELA local search features	ela_local.fun_evals.sd	aggregations (standard deviation) of the performed local searches
information content features	ic.eps.ratio	ratio of partial information sensitivity where the ratio is 0.5
cell mapping features	cm_angle.dist_ctr2worst.mean	arithmetic mean of distances from the cell center to the worst observation within the cell (over all cells)
cell mapping features	cm_angle.costs_runtime	runtime (in seconds) needed for the computation of these features
cell mapping features	cm_grad.mean	arithmetic mean of the aforementioned lengths
linear model features	limo.avg_length.norm	length of the average coefficient vector (based on normalized vectors)
linear model features	limo.cor.norm	correlation of all coefficient vectors (based on normalized vectors)
linear model features	limo.sd_ratio.reg	max-by-min ratio of the standard deviations of the (non-intercept) coefficients (based on regular ones)
principal component features	pca.expl_var.cov_init	proportion of the explained variance when applying PCA to the covariance matrix of the entire initial design (init)
principal component features	pca.expl_var_PC1.cov_x	proportion of variance which is explained by the first principal component when applying PCA to the covariance matrix of the decision space (x)
principal component features	pca.expl_var_PC1.cov_init	proportion of variance which is explained by the first principal component when applying PCA to the covariance matrix of the entire initial design

**Table 5:** The accuracy of classifying BBOB instances into 24 problem categories using different ELA features for D = 2, D = 5, D = 10 and D = 20. The first row shows the accuracy of the feature set (19 ELA features) after feature selection to classify the BBOB instances into 24 problem categories. Three comparison experiments are set up.

		D = 2	D = 5	D = 10	D = 20
	Feature selection ( 19 features ) Classifier: CNN	99.97%	98.78%	98.66%	98.13%
Comparative Experiment 1	Feature selection ( 19 features ) Classifier: SVM	97.5%	96.4%	95.3%	93.18%
	Feature selection ( 19 features ) Classifier: RF	96.35%	95.21%	94.74%	94.8%
Comparative Experiment 2	Random selection ( 19 features )	50.33%	62.88%	74.17%	55.33%
	Initial feature set ( 169 features )	99.97%	96.78%	95.71%	93.79%
Comparative Experiment 3	2D-Feature selection (14 features)	99.97%	−	−	−
	5D-Feature selection (22 features)	−	99.56%	−	−
	10D-Feature selection (13 features)	−	−	99.22%	−
	20D-Feature selection (21 features)	−	−	−	99.22%

Comparison Experiment 1 shows the accuracy results of classifying the set of 19 features after feature selection by Support Vector Machine, Random Forest Classifier (Rows 2 and 3 in the table). Comparison Experiment 2 is the accuracy result of classifying BBOB instances into 24 problem categories based on the randomly selected feature set (19 features) and the initial feature set (169 features) (Rows 4 and 5 in the table). Comparison Experiment 3 is the accuracy of the feature set (14, 22, 13, 21 features) obtained after feature selection to classify the BBOB instances into 24 problem categories with D = 2, 5, 10, 20 (Rows 6, 7, 8, and 9 in the table).

**Table 6:** Results of algorithm selection strategy for 24 BBOB function selection algorithms. The gray color indicates that the algorithm is selected and the white color indicates that the algorithm is not selected. If there are multiple algorithms selected for the obtained function, one algorithm is randomly selected from the selected algorithm as the recommended algorithm.

	F1	F2	F3	F4	F5	F6	F7	F8	F9	F10	F11	F12
CoDE												
CMA-ES												
SSA												
L-SHADE												
ZOA												
	F13	F14	F15	F16	F17	F18	F19	F20	F21	F22	F23	F24
CODE												
CMAES												
SSA												
L-shade												
ZOA												

**Table 7:** In D = 10, the rank results of CNN-HT approach and comparison algorithms. In the table, (+), (−) and (=) indicate that the corresponding algorithms are significantly worse than CNN-HT, significantly better than CNN-HT, and not significantly different from CNN-HT by the hypothesis testing method on the corresponding problems, respectively.

	CNN-HT	PAP	CoDE	CMA-ES	SSA	L-SHADE	ZOA
f1	1	1 (=)	1 (=)	1 (=)	1 (=)	1 (=)	1 (=)
f2	1	1 (=)	1 (=)	1 (=)	1 (=)	1 (=)	1 (=)
f3	1	1 (=)	1 (=)	6 (+)	1 (=)	1 (=)	6 (+)
f4	1	1 (=)	1 (=)	6 (+)	1 (=)	1 (=)	6 (+)
f5	1	1 (=)	1 (=)	1 (=)	1 (=)	1 (=)	1 (=)
f6	1	1 (=)	1 (=)	1 (=)	1 (=)	1 (=)	1 (=)
f7	1	1 (=)	1 (=)	6 (+)	1 (=)	1 (=)	6 (+)
f8	1	3 (=)	1 (=)	3 (=)	7 (+)	3 (=)	6 (+)
f9	1	7 (+)	3 (=)	1 (=)	5 (+)	4 (=)	6 (+)
f10	1	6 (+)	4 (=)	3 (=)	7 (+)	1 (=)	5 (+)
f11	2	1 (=)	5 (+)	3 (=)	4 (=)	5 (+)	5 (+)
f12	1	3 (=)	7 (+)	1 (=)	5 (+)	3 (=)	5 (+)
f13	1	1 (=)	1 (=)	1 (=)	5 (+)	5 (+)	5 (+)
f14	1	1 (=)	5 (+)	1 (=)	7 (+)	5 (+)	1 (=)
f15	1	3 (+)	4 (+)	4 (+)	4 (+)	4 (+)	1 (=)
f16	2	2 (=)	5 (+)	5 (+)	2 (=)	5 (+)	1 (=)
f17	1	1 (=)	1 (=)	6 (+)	5 (+)	1 (=)	7 (+)
f18	1	1 (=)	1 (=)	5 (+)	7 (+)	1 (=)	5 (+)
f19	1	6 (+)	3 (+)	4 (+)	5 (+)	6 (+)	1 (=)
f20	2	3 (=)	1 (=)	5 (+)	3 (=)	5 (+)	5 (+)
f21	3	1 (=)	2 (=)	7 (+)	5 (=)	6 (=)	4 (=)
f22	3	1 (=)	4 (=)	7 (+)	6 (+)	4 (=)	2 (=)
f23	1	3 (+)	4 (+)	4 (+)	4 (+)	4 (+)	1 (=)
f24	1	3 (+)	4 (+)	4 (+)	4 (+)	4 (+)	1 (=)
average	1.291	2.208	2.583	3.583	3.833	3.041	3.458
	**PAP**	**CoDE**	**CMA-ES**	**SSA**	**L-SHADE**	**ZOA**	
Significantly worse than CNN-HT	5	8	13	13	9	12	

**Table 8:** In D = 2, the rank results of CNN-HT approach and comparison algorithms. In the table, (+), (−) and (=) indicate that the corresponding algorithms are significantly worse than CNN-HT, significantly better than CNN-HT, and not significantly different from CNN-HT by the hypothesis testing method on the corresponding problems, respectively.

	CNN-HT	PAP	CoDE	CMA-ES	SSA	L-SHADE	ZOA
f1	1	1 (=)	1 (=)	1 (=)	1 (=)	1 (=)	1 (=)
f2	1	1 (=)	1 (=)	1 (=)	1 (=)	1 (=)	1 (=)
f3	1	1 (=)	1 (=)	6 (+)	1 (=)	1 (=)	6 (+)
f4	1	1 (=)	1 (=)	6 (+)	1 (=)	1 (=)	6 (+)
f5	1	1 (=)	1 (=)	1 (=)	1 (=)	1 (=)	1 (=)
f6	1	1 (=)	1 (=)	1 (=)	1 (=)	1 (=)	1 (=)
f7	3	1 (=)	1 (=)	6 (+)	6 (+)	3 (=)	3 (=)
f8	1	1 (=)	1 (=)	1 (=)	7 (+)	1 (=)	1 (=)
f9	1	7 (+)	3 (=)	1 (=)	6 (+)	5 (=)	3 (=)
f10	1	1 (=)	5 (=)	1 (=)	7 (+)	1 (=)	6 (+)
f11	2	2 (=)	5 (+)	1 (=)	4 (=)	5 (+)	5 (+)
f12	2	3 (=)	6 (+)	1 (=)	6 (+)	3 (=)	3 (=)
f13	1	1 (=)	1 (=)	1 (=)	6 (+)	5 (=)	6 (+)
f14	1	1 (=)	5 (+)	1 (=)	7 (+)	5 (+)	1 (=)
f15	2	3 (+)	5 (+)	6 (+)	7 (+)	4 (+)	1 (=)
f16	2	2 (=)	6 (+)	7 (+)	4 (=)	5 (+)	1 (=)
f17	1	1 (=)	1 (=)	5 (+)	5 (+)	1 (=)	5 (+)
f18	1	3 (=)	4 (=)	6 (+)	7 (+)	1 (=)	5 (=)
f19	1	6 (+)	4 (+)	4 (+)	3 (+)	6 (+)	1 (=)
f20	2	2 (=)	1 (−)	5 (+)	3 (=)	5 (+)	5 (+)
f21	2	1 (=)	4 (=)	7 (+)	2 (=)	4 (=)	4 (=)
f22	2	1 (=)	5 (=)	6 (+)	6 (+)	4 (=)	2 (=)
f23	2	2 (=)	5 (+)	1 (−)	5 (+)	5 (+)	2 (=)
f24	1	3 (+)	4 (+)	4 (+)	4 (+)	4 (+)	1 (=)
average	1.416	1.958	3	3.33	4.208	3.0416	2.958
	**PAP**	**CoDE**	**CMA-ES**	**SSA**	**L-SHADE**	**ZOA**	
Significantly worse than CNN-HT	4	8	12	14	8	7	

**Table 9:** In D = 5, the rank results of CNN-HT approach and comparison algorithms. In the table, (+), (−) and (=) indicate that the corresponding algorithms are significantly worse than CNN-HT, significantly better than CNN-HT, and not significantly different from CNN-HT by the hypothesis testing method on the corresponding problems, respectively.

	CNN-HT	PAP	CoDE	CMA-ES	SSA	L-SHADE	ZOA
f1	1	1 (=)	1 (=)	1 (=)	1 (=)	1	1 (=)
f2	1	1 (=)	1 (=)	1 (=)	1 (=)	1 (=)	1 (=)
f3	1	1 (=)	1 (=)	6 (+)	1 (=)	1 (=)	6 (+)
f4	1	1 (=)	1 (=)	6 (+)	1 (=)	1 (=)	6 (+)
f5	1	1 (=)	1 (=)	1 (=)	1 (=)	1 (=)	1 (=)
f6	1	1 (=)	1 (=)	1 (=)	1 (=)	1 (=)	1 (=)
f7	1	1 (=)	1 (=)	6 (+)	1 (=)	1 (=)	7 (+)
f8	1	3 (=)	1 (=)	3 (=)	6 (+)	3 (=)	6 (+)
f9	1	7 (+)	4 (=)	2 (=)	6 (+)	3 (=)	5 (+)
f10	2	6 (+)	4 (=)	3 (=)	7 (+)	1 (=)	5 (+)
f11	1	1 (=)	5 (+)	1 (=)	5 (+)	1 (=)	5 (+)
f12	1	3 (=)	7 (+)	1 (=)	5 (+)	3 (=)	5 (+)
f13	1	2 (=)	4 (=)	3 (=)	5 (+)	5 (+)	5 (+)
f14	1	4 (=)	5 (=)	1 (=)	6 (+)	6 (+)	1 (=)
f15	1	3 (=)	3 (=)	6 (+)	6 (+)	3 (=)	1 (=)
f16	2	2 (=)	5 (+)	5 (+)	2 (=)	5 (+)	1 (=)
f17	1	1 (=)	1 (=)	5 (+)	5 (+)	1 (=)	5 (+)
f18	1	1 (=)	1 (=)	5 (+)	7 (+)	1 (=)	5 (+)
f19	1	6 (+)	7 (+)	4 (+)	5 (+)	3 (+)	1 (=)
f20	3	3 (=)	2 (=)	5 (+)	1 (=)	5 (+)	5 (+)
f21	3	1 (=)	4 (=)	7 (+)	6 (=)	4 (=)	1 (=)
f22	3	1 (=)	4 (=)	7 (+)	6 (+)	4 (=)	2 (=)
f23	1	3 (+)	4 (+)	4 (+)	4 (+)	4 (+)	1 (=)
f24	1	3 (+)	4 (+)	4 (+)	4 (+)	4 (+)	1 (=)
average	1.333	2.375	3	3.667	3.875	2.625	3.25
	**PAP**	**CoDE**	**CMA-ES**	**SSA**	**L-SHADE**	**ZOA**	
Significantly worse than CNN-HT	5	6	13	14	7	12	

**Table 10:** In D = 20, the rank results of CNN-HT approach and comparison algorithms. In the table, (+), (−) and (=) indicate that the corresponding algorithms are significantly worse than CNN-HT, significantly better than CNN-HT, and not significantly different from CNN-HT by the hypothesis testing method on the corresponding problems, respectively.

	CNN-HT	PAP	CoDE	CMA-ES	SSA	L-SHADE	ZOA
f1	1	1 (=)	1 (=)	1 (=)	1 (=)	1 (=)	1 (=)
f2	1	1 (=)	1 (=)	1 (=)	1 (=)	1 (=)	1 (=)
f3	1	1 (=)	1 (=)	6 (+)	1 (=)	1 (=)	6 (+)
f4	1	1 (=)	1 (=)	6 (+)	1 (=)	1 (=)	6 (+)
f5	1	1 (=)	1 (=)	1 (=)	1 (=)	1 (=)	1 (=)
f6	1	1 (=)	1 (=)	1 (=)	1 (=)	1 (=)	1 (=)
f7	1	1 (=)	1 (=)	6 (+)	1 (=)	1 (=)	6 (+)
f8	3	1 (−)	3 (=)	1 (−)	6 (+)	3 (=)	7 (+)
f9	1	5 (+)	3 (=)	1 (=)	5 (+)	3 (=)	5 (+)
f10	1	4 (+)	4 (+)	1 (=)	4 (+)	1 (=)	4 (+)
f11	1	1 (=)	4 (+)	1 (=)	4 (+)	4 (+)	4 (+)
f12	3	2 (=)	6 (+)	1 (=)	5 (+)	7 (+)	4 (+)
f13	1	1 (=)	1 (=)	1 (=)	5 (+)	5 (+)	5 (+)
f14	1	1 (=)	5 (+)	3 (=)	5 (+)	7 (+)	3 (=)
f15	1	3 (+)	3 (+)	3 (+)	3 (+)	3 (+)	1 (=)
f16	2	2 (=)	5 (+)	5 (+)	2 (=)	5 (+)	1 (=)
f17	1	1 (=)	1 (=)	5 (+)	5 (+)	1 (=)	5 (+)
f18	1	1 (=)	1 (=)	5 (+)	7 (+)	4 (=)	5 (+)
f19	2	4 (+)	3 (+)	4 (+)	6 (+)	6 (+)	1 (=)
f20	2	2 (=)	1 (=)	5 (+)	2 (=)	7 (+)	5 (+)
f21	3	1 (=)	4 (=)	7 (+)	5 (=)	6 (=)	1 (=)
f22	2	5 (+)	4 (=)	6 (+)	6 (+)	1 (=)	2 (=)
f23	1	1 (=)	4 (+)	4 (+)	4 (+)	4 (+)	1 (=)
f24	1	3 (+)	3 (+)	3 (+)	3 (+)	3 (+)	1 (=)
average	1.416	1.875	2.583	3.25	3.5	3.208	3.208
	**PAP**	**CoDE**	**CMA-ES**	**SSA**	**L-SHADE**	**ZOA**	
Significantly worse than CNN-HT	6	9	13	14	10	12	

**Table 11:** In D = 10, the results of ranking the CNN-HT method and the comparison algorithms in different sets of algorithms. In the table, (+), (−) and (=) indicate that the corresponding algorithms are significantly worse than CNN-HT, significantly better than CNN-HT, and not significantly different from CNN-HT by the hypothesis testing method on the corresponding problems, respectively.

	CNN-HT	PAP	GL25	CMAES	ABC	SaDE
f1	1	1 (=)	1 (=)	1 (=)	1 (=)	1 (=)
f2	1	1 (=)	1 (=)	1 (=)	1 (=)	1 (=)
f3	1	1 (=)	4 (+)	4 (+)	4 (+)	1 (=)
f4	1	1 (=)	4 (+)	4 (+)	4 (+)	3 (+)
f5	1	1 (=)	1 (=)	1 (=)	1 (=)	1 (=)
f6	1	1 (=)	5 (+)	1 (=)	6 (+)	1 (=)
f7	1	2 (=)	4 (+)	6 (+)	5 (+)	2 (=)
f8	1	3 (+)	5 (+)	2 (=)	5 (+)	4 (+)
f9	1	1 (=)	5 (+)	1 (=)	5 (+)	4 (+)
f10	1	1 (=)	4 (+)	1 (=)	4 (+)	4 (+)
f11	1	1 (=)	4 (+)	1 (=)	4 (+)	4 (+)
f12	1	1 (=)	4 (+)	1 (=)	4 (+)	4 (+)
f13	1	3 (=)	4 (+)	1 (=)	4 (+)	4 (+)
f14	1	1 (=)	5 (+)	1 (=)	5 (+)	4 (+)
f15	1	1 (=)	1 (=)	5 (+)	6 (+)	1 (=)
f16	2	3 (=)	5 (+)	3 (=)	5 (+)	1 (=)
f17	2	2 (=)	4 (=)	5 (+)	5 (+)	1 (=)
f18	3	4 (+)	1 (=)	4 (+)	4 (+)	2 (=)
f19	1	3 (+)	5 (+)	1 (=)	5 (+)	3 (+)
f20	1	1 (=)	4 (+)	4 (+)	4 (+)	1 (=)
f21	1	4 (=)	1 (=)	6 (+)	4 (=)	1 (=)
f22	1	3 (=)	3 (=)	6 (+)	5 (=)	1 (=)
f23	1	1 (=)	4 (+)	1 (=)	4 (+)	4 (+)
f24	3	1 (=)	5 (+)	1 (=)	6 (+)	4 (=)
average	1.25	1.75	3.5	2.583	4.208	2.375
	**PAP**	**GL25**	**CMAES**	**ABC**	**SaDE**	
Significantly worse than CNN-HT	3	16	9	19	10	

## Data Availability

Data are contained within the article.
